# CFD Modeling of Primary Breakup in an EIGA Atomizer for Titanium Alloy Powder Production

**DOI:** 10.3390/ma16175900

**Published:** 2023-08-29

**Authors:** Kuaikuai Guo, Changsheng Liu, Wei Chen, Chang Luo, Jianzhong Li

**Affiliations:** 1 School of Metallurgy, Northeastern University, Shenyang 110819, China; guokuaikuai@mail.neu.edu.cn; 2 School of Materials Science and Engineering, Northeastern University, Shenyang 110819, China; 3 Baosteel Roll Science & Technology Co., Ltd., Changzhou 213019, China

**Keywords:** numerical simulation, titanium alloy metal powder, gas atomization, primary breakup

## Abstract

Electrode induction melting gas atomization (EIGA) technology is a commonly used and effective method for producing spherical metal powders in additive manufacturing. In this paper, we aim to describe the atomization and fragmentation of liquid sheets from a typical swirl nozzle and highlight the primary breakup of titanium alloy powder production. We developed a computational fluid dynamics (CFD) approach to simulate the primary disintegration process of the molten metal using the volume of fluid (VOF) method coupled with the large eddy simulation turbulence model (LES). Our numerical results show that high-speed spraying creates supersonic airflow in the atomization chamber. Recirculation is the main area where primary atomization occurs. The formation of the recirculation zone is the direct driving force that allows atomization to proceed, which will increase turbulence intensity and achieve higher atomization efficiency. VOF-LES simulation can capture some qualitative results such as conical melt-sheet shape, wave formation, ligament formation, and perforation formation. The primary droplet size mainly ranges between 200 and 800 μm. Finally, with increasing gas pressure, the particle size of the atomized powder gradually decreases, and the particle size distribution becomes narrower.

## 1. Introduction

Laser 3D printing titanium alloy techniques have strict requirements for the chemical composition, sphericity, fluidity, and particle size distribution of titanium alloy spherical powder [1,2,3,4]. Titanium alloy powders are distinct from traditional metallic powders, such as iron-based stainless steel, nickel-based superalloys, and cobalt-based alloys [5]. The robust absorption abilities of titanium alloy and its excessive temperature response make the preparation process for titanium alloy powder unique from conventional metallic powder. Gas atomization is a method for preparing high-temperature metal powder for 3D printing that uses gas impact with high kinetic energy to break up the metal melt into fine droplets [6,7,8]. This process generally consists of two essential stages [9,10]: Firstly, primary atomization refers to the procedure where high-temperature molten metal flowing from the delivery tube interacts with the high-speed gas and disperses to form a liquid film, which is then broken into initial droplets [11]; secondly, secondary atomization refers to the procedure where the initial droplets are broken into finer droplets by the airflow wrapped to the turbulent layer. Moreover, primary atomization is the dominating process [12].

The system being studied is a particular free-fall atomization process called electrode induction melting gas atomization (EIGA) [8,13,14]. This process uses a prealloyed bar as the electrode and induction heating without a crucible, which is atomized by inert gas. The high-temperature melt atomized by EIGA is not directly contacted by the delivery tube, resulting in a more media-like connection channel. The EIGA process achieves such high purity that it has become the preferred method for preparing titanium alloy powder. Atomization is a complicated physicochemical exchange process [15,16]. Traditional experimental methods present significant challenges in characterizing the complete atomization method, as well as demonstrating the impact of fragmentation on liquid droplets. In general, numerical simulations are mixed with the idea of finite tissue or volumes. Streamlined techniques of computational fluid dynamics can achieve the melted metal’s gas trajectory, flow field structure, and fragmentation process [17,18,19].

Phenomena such as the breakup process of close-coupled gas atomization have been the subject of some studies. However, this research has focused on the vacuum induction melting gas atomization (VIGA) process and are mostly concerned with studying the effects of processing parameters on powder particle size. Only a limited number of studies have reported on the complete modeling of the free fall nozzle EIGA breakup process in the literature. Table 1 compares atomization models in previous literature.

In this paper, a powder production route using EIGA is investigated. The Euler–Euler method, volume of fluid (VOF) analysis, multi-phase flow model, and large eddy simulation (LES) model [10] were adopted to simulate primary atomization in the recirculation zone. In addition, the influence of gas pressure on the formation mechanism and powder particle size is discussed. This research will lead to the industry’s better understanding of the crushing mechanism and optimizing process parameters to improve EIGA atomization efficiency.

## 2. Materials and Methods

### 2.1. EIGA Atomization Tower

**Figure 1:**
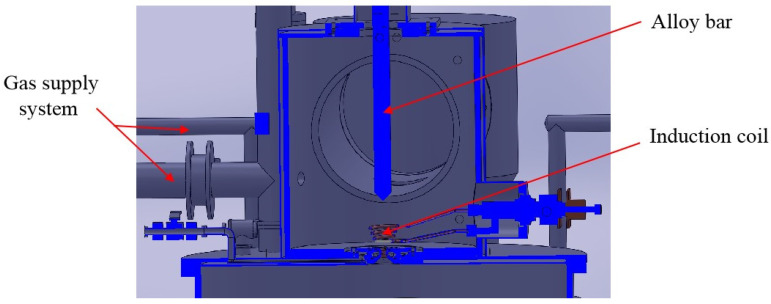
The structure diagram of smelting chamber.

The EIGA atomization tower consists of a vacuum induction melting and gas atomization system. Figure 1 shows a cross-section of the melting chamber, which consists of a gas supply system, an alloy bar feed system, and an induction coil.

**Figure 2:**
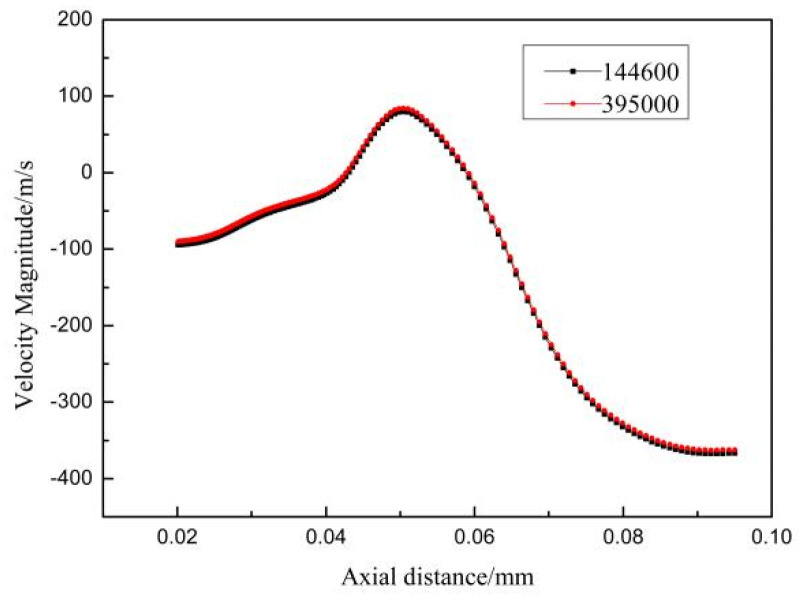
Influence of the mesh grid number on gas velocity along the axial line.

Considering the atomization process, the flow field was axisymmetrically distributed to improve calculation efficiency with the nozzle axis as the centerline. Therefore, the actual center-symmetric three-dimensional (2D) structure was simplified to a two-dimensional structure. The dimensions of the 2D axisymmetric geometry were 50 mm long in the radial direction and 138 mm long in the axial direction. The total mesh number was 395,000. Figure 2 shows the single-phase simulation calculated at different mesh numbers under an atomization pressure of 5 MPa. The mesh number had minor effects on gas velocity along the axial line, verifying that the independence of the mesh.

**Figure 3:**
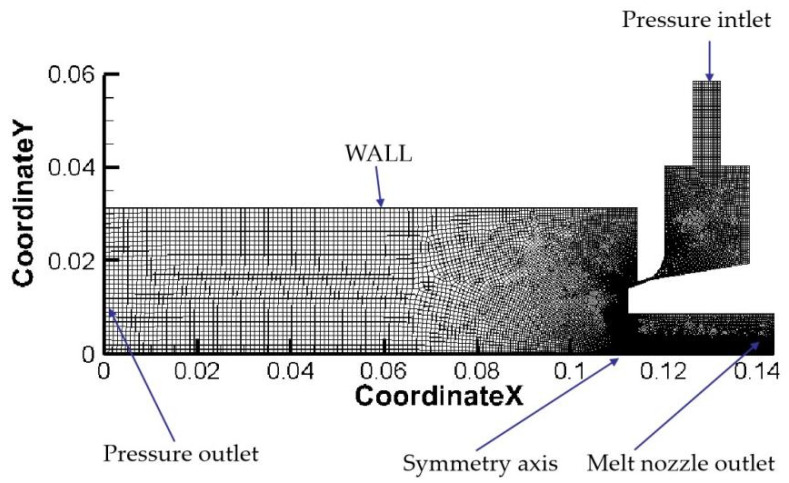
Plot of mesh structure and boundary conditions of 1/2 EIGA model.

A mapping method was used to divide the non-structural mesh. The mesh could be refined or coarsened by ANSYS-Fluent 19 Fluent automatically, as shown in Figure 3. Since Fluent requires the X-axis to be the rotation axis of the axis-symmetric model, the symmetry axis boundary of the geometric model was set as the X-axis and the rotation axis boundary. The pressure inlet boundary was chosen as the gas inlet, and the upper and left boundaries of the calculation area were set as the pressure outlet. The melt had a certain velocity under the effect of gravity through the delivery tube, so the delivery tube inlet was selected as the mass flow inlet. The metal walls on both sides of the atomization chamber were defined as the Wall.

### 2.2. Modeling the Discontinuous Droplets Primary Breakup Process

A numerical model was developed in CFD software ANSYS-Fluent 2020R1 to describe the gas–liquid interaction in the flow field. We adopted the VOF model [23] for multi-phase flow based on the Eulerian method, which can effectively calculate the fluid state under gas–liquid interaction conditions. If the cell is filled with liquid, the volume fraction is 1, whereas if the grid cell is filled with gas, then its value is 0. At the gas–liquid interface, the volume fraction varies from 1 to 0. The VOF multi-phase model is controlled by Equation (1). Other physical properties such as density in each cell is calculated using weighted averages, as shown in Equation (2).

(1) $$\frac{1}{\rho_{q}}\left[\frac{\partial }{\partial t}\left(\alpha_{q}\rho_{q}\right)+\nabla \cdot \left(\alpha_{q}\rho_{q}U_{q}\right)\right]=0$$

(2) $$\rho =\alpha_{2}\rho_{2}+\left(1-\alpha_{2}\right)\rho_{1}$$

where *U_q_* is velocity, the *α_q_* is volume fraction, and the *ρ_q_* is density.

The gas jet velocity of the nozzle exit is greater than 5 MPa, so the compressibility of gas jet should be considered. Compressible Navier–Stokes (N–S) equations control the flow of the two-phase gas flow. Hence, the continuity equation and momentum equation are expressed by Equations (3) and (4), respectively:

(3) $$\frac{\partial }{\partial t}\left(\alpha \rho \right)+\nabla \cdot \left(\alpha \rho \mu \right)=0$$

(4) $$\frac{\partial }{\partial t}\left(\rho \mu \right)+\nabla \cdot \left(\rho \mu \mu \right)=-\nabla P+\nabla \cdot \left[\mu \left(\nabla_{\mu }+\nabla \mu^{T}\right)\right]+\rho g+F\;$$

where *u*, *P*, *ρ*, *μ*, *α*, and *F* are the velocity, pressure, density, viscosity, surface tension, and indicator fields, respectively.

The LES model [24,25] is used to calculate the turbulent flow in the atomization process and can demonstrate the transient change process of the turbulent flow more accurately. The melt temperature and diameter of the liquid metal were set to 2193.15 k and 4 mm, respectively. The gas atomization pressure ranged between 5.0 and 7.0 Mpa.

The solver uses the pressure-based transient method of simulation. The time step was adaptive, and the initial step was 1 × 10^−6^ s. The properties of the argon and titanium liquid states are shown in Table 2. The gravitational acceleration was 9.8 m/s^2^ along the *Y*-axis.

## 3. Results and Discussion

### 3.1. Single-phase Flow Field

**Figure 4:**
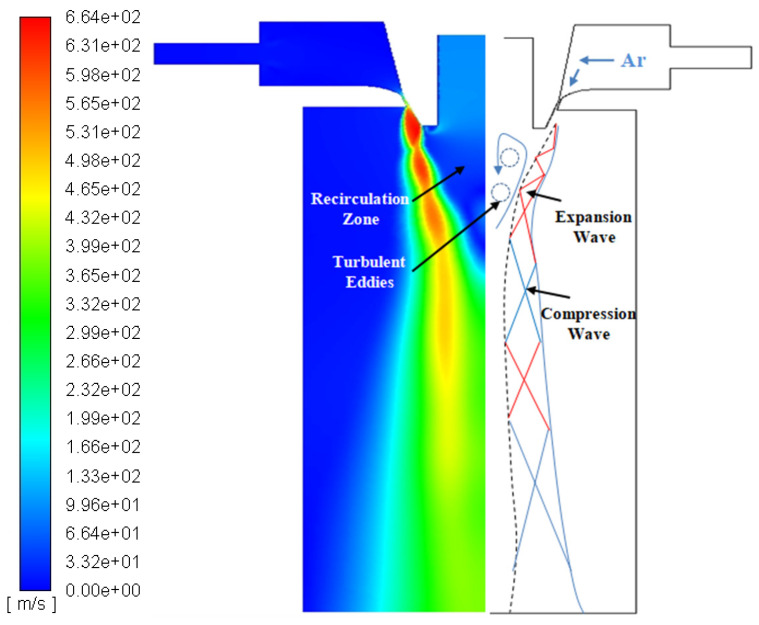
Velocity contour of single-phase flow field (m/s).

Figure 4 depicts the velocity contour of a single-phase flow field. It can be observed that the gas stream flows at a low velocity (<50 m/s) within the gas chamber. At the exit of the annular slit nozzle, the high-pressure gas flow is compressed by the contracted part of the slit to form a compressed flow. The pressure differential causes an expansion wave cluster to form at the nozzle end. Furthermore, the velocity increases and the pressure decreases, forming a supersonic airflow with a maximum velocity of 660 m/s. The increased gas pressure, however, causes the expansion wave cluster to converge into a compression wave after reflecting from the free boundary. The airflow velocity decreases, and the pressure increases at the compression wave. This phenomenon causes the airflow to accelerate and decelerate during the downward flow, creating a “thick chain-shaped” velocity cloud.

The fluid’s path into the atomization chamber encounters numerous surfaces at various speeds as it travels through the chamber. The intermittent surface is extremely unstable and generates vortices. Additionally, the vortex will be involved in the surrounding fluid, which flows into the jet. Turbulent eddies in the recirculation result in the gas jet deforming and developing on both sides of the axis, forming a free turbulent mixing layer. Consequently, the reflections of the expansion and compression waves are limited to the sonic boundary inside the jet and gradually disappear as the propagation distance increases.

**Figure 5:**
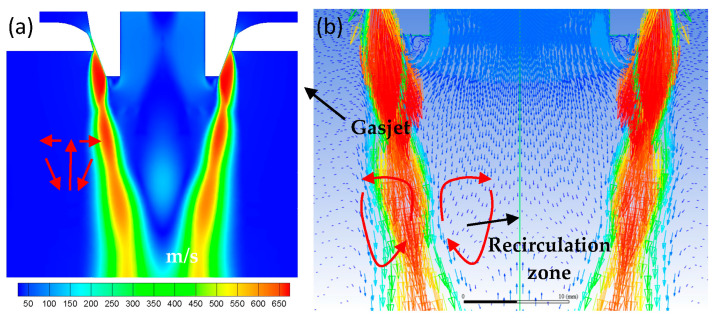
(**a**) Velocity contour of recirculation zone; (**b**) velocity vector diagram (m/s).

Figure 5 exhibits the recirculation zone’s morphological cloud diagram and velocity vector diagram. Figure 5a depicts an inverted conical region at the bottom of the delivery tube where the flow direction is opposite to the inlet flow. This area is called the recirculation zone. The stagnation point at the end of the recirculation is the point where the velocity is zero. Gas disturbances cause the formation of the recirculation zone. A series of turbulent vortices on the inside of the free boundary form when high-pressure gas enters the chamber and create a high-speed jet, (Figure 5b). The presence of these vortices causes the gas to move forward with rotational momentum. The propagation areas of the turbulent vortices are expanded with the continuous injection of the gas jet and eventually converge to form the recirculation zone. In summary, the formation of the recirculation zone is the direct driving force permitting atomization to proceed. The dimensions and location of the recirculation area also indirectly impact the effectiveness of atomization.

The pressure state in the recirculation zone is crucial for obtaining suction in the molten metal flow, i.e., it significantly influences the stability of the atomization process. The difference in pressure between the bottom of the delivery tube and the furnace is known as suction pressure.

(5) $$\Delta P=P_{d\;}-{\;P}_{a}$$

where *P_d_* is the pressure at the bottom of the delivery tube; *P_a_* is the pressure of the atomization chamber. The negative suction force of the molten metal to the atomization chamber encourages the steady flow of molten metal into the delivery tube. Positive suction pressure stops Tte flow of molten metal in the delivery tube, resulting in the obstruction of the delivery tube.

**Figure 6:**
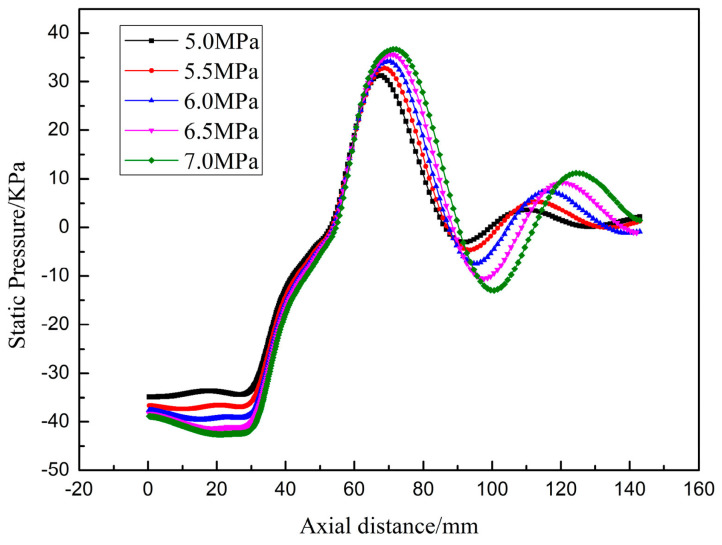
Static pressure distribution along the centerline of the exit slit of the nozzle at five different pressures.

Figure 6 shows the static pressure distribution along the centerline of the exit slit of the nozzle. To quantify the suction pressure, Table 3 compares the static pressure values at the nozzle outlet. When the pressure increases from 5.5 to 7.0 MPa, the increase in suction pressure is not significant. In the direction away from the delivery tube, the static pressure value first decreases, then gradually increases, i.e., the suction pressure first increases and then decreases. The process of increasing suction pressure ensures the continuous flow of molten metal. The pressure near the end of the recirculation zone, known as stagnation pressure, is significant, and a necessary condition for recirculation formation. Ting et al. [26] claimed that the stagnation pressure in the recirculation zone affects how much gas goes into it. The higher the pressure, the more gas there is at the stagnation point, suggesting that more gas goes into the recirculation. When the gas pressure is 7.0 MPa, the pressure fluctuation downstream of the recirculation can reach 20 KPa. Ultimately, the atomized powder will likely move to the atomization zone and mix with the molten droplets that have not solidified yet to form satellite balls, affecting the quality of the prepared alloy powder.

### 3.2. Gas−Liquid Two-Phase Flow Field

**Figure 7:**
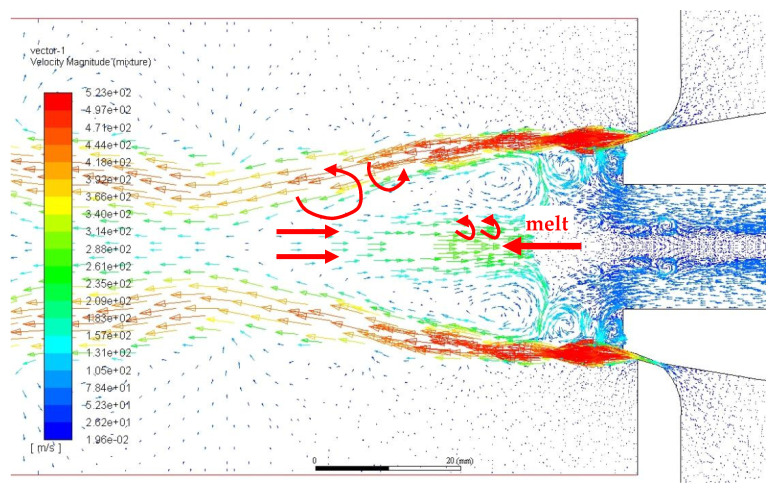
Velocity vector diagram of two phase flow field (m/s).

We compared the single- (gas) and two-phase flow fields (gas and liquid) flow fields and observed extra turbulence in the flow state of the recirculation zone in the two-phase flow field (Figure 7). The observed increase in air velocity within the recirculation zone was accompanied by a significant increase and enhancement in turbulence within the free boundary. Even a tiny portion of the vortex can manifest itself in the delivery tube. It can be wrapped in molten metal to accelerate the flow to the recirculation zone. In turn, it will be rapidly broken by the vortex. Although the droplet-breaking characteristics inside the free boundary are difficult to observe in industrial production, the simulation analysis of the two-phase flow field clearly revealed the flow traits of the melt.

**Figure 8:**
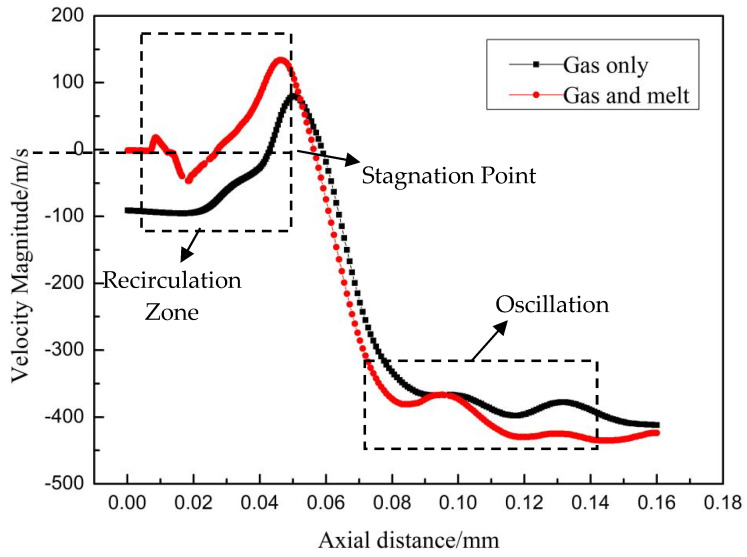
Axial velocity distribution along center axis.

As described in the previous section, there is a recirculation zone below the inflow tube. Figure 8 shows the velocity change curve on the center line of the flow field. Initially, the airflow velocity value increased, then decreased, and the recirculation zone length was about 40 mm below the stagnation point. In addition, the velocity direction was downward, and the velocity gradually increased (maximum 453 m/s). The velocity stagnation points in both single-phase and two-phase flow conditions are located at the downstream end of the recirculation zone. The velocity variation in two-phase flow conditions is greater due to the introduction of high-temperature melt, which increases jet turbulence. The velocity oscillation observed at the end of the main jet zone is attributed to the coherent effect of expansion waves. The gas expansion and compression at the inner side of the sonic boundary has the opposite effect on gas flow at the axial position because the gas velocity inside the sonic boundary on both sides changes in opposite directions, causing violent velocity fluctuations.

### 3.3. Mechanisms of Droplet Formation in Primary Atomization Process

**Figure 9:**
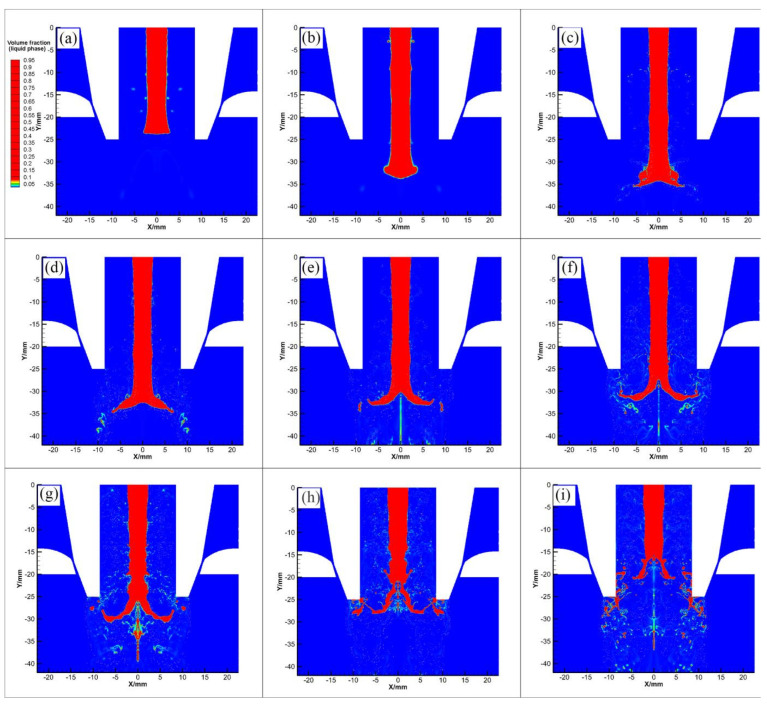
Fragmentation morphology of high-temperature melts in different stages of primary atomization (**a**) Time = 0.015 s; (**b**) Time = 0.020 s; (**c**) Time = 0.026 s; (**d**) Time = 0.0264 s; (**e**) Time = 0.0268 s; (**f**) Time = 0.0270 s; (**g**) Time = 0.0282 s; (**h**) Time = 0.0290 s; (**i**) Time = 0.0304 s.

Figure 9 illustrates the fragmentation morphology of high-temperature melts in a free-fall nozzle. At a rate of 0.015 s, the metal liquid flowed from the delivery tube into the atomization chamber. Under its own momentum in the recirculation zone, the liquid film began to reduce the flow speed or stop flowing. As the droplet continued to fall, the molten metal started to deform and expand in a radial direction (Figure 9b). As seen in Figure 9c,d, the metal liquid flow had broken while the end of the columnar liquid flow laterally expanded the liquid membrane. The liquid film had deformed and twisted upward. As a result, the excitation caused melting on both sides of the liquid film to break up into an umbrella-like daisy, known as the “umbrella mechanism” (Figure 9e). The subsequent stretching and thinning of the film resulted in the continuous emergence of large droplets from the edge of the film (Figure 9f). Figure 9h shows that the melt flowing into the recirculation zone is completely peeled off into a liquid film. This film is constantly growing to form a thin and elongated liquid band. In Figure 9i, this non-stationary liquid band is very short and breaks into large molten droplets quickly. This type of droplet is a transitional form between a liquid film and large droplets. The liquid band was completely broken at 0.03028 s, and the large droplets were further broken to form smaller droplets. The atomization procedure involves a continual exchange of the kinetic energy of gas and the surface energy of droplets. The high-speed gas jet transports heat energy from the molten droplets at high temperatures as they spheroidize under surface tension and turn into a spherical powder.

**Figure 10:**
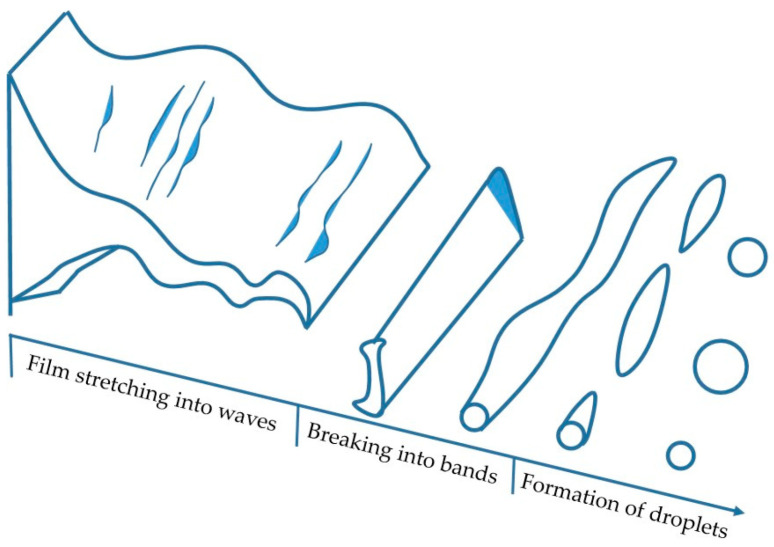
Mode of droplet breakup.

The most widely accepted theory of fragmentation was proposed by Rayleigh, who argued that disturbances inevitably occur in a gas flow field when there are disturbances in the liquid stream [27]. The increasing amplitude of the disturbance will result in the liquid jet’s destabilization and fragmentation into droplets. Based on Rayleigh’s study, Dombrowski et al. [28] proposed a liquid flow fragmentation model in which the liquid film gradually extends and breaks into droplets as the perturbation wave grows, as shown in Figure 10. The molten metal stream is first extended into a liquid film by airflow perturbation. The film progresses into a wave, then a half wave, and finally a band. The film’s thickness and wavelength determine the band’s length and width.

(6) $$D_{L}=3\sqrt{\lambda s}$$

where *λ* is the wavelength; *s* is the liquid film thickness.

However, under high-speed airflow, the liquid band formed by liquid film fragmentation becomes extremely unstable and rapidly divides into short waves. Under the action of shock oscillations, it is further broken into initial droplets. The diameter of the initial droplet is calculated by [29]:

(7) $$d_{p}=1.88d_{L}{\left(1+3\mathrm{Oh}\right)}^{1/6}$$

where the Oh number is a dimensionless number used in fluid mechanics to measure viscous forces and surface tension (σ). Its formula is Oh = μ_l_/(ρ_l_σ*d_L_*)1/2, where μ_l_ is the drop viscosity and ρ_l_ is the droplet material density. Since the Oh number is too small, the equation can be simplified to:

(8) $$d_{p}=1.88d_{L}$$

**Figure 11:**
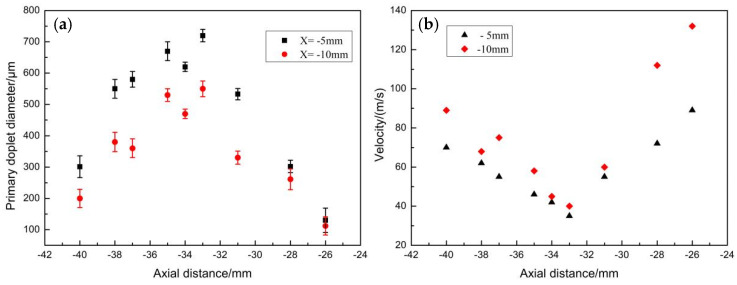
Velocity and diameter distributions of the parent droplets. (**a**) Droplet diameters (μm) at positions X = −5 mm and X = −10 mm; (**b**) velocity (m/s) at position X = −5 mm and X = −10 mm lines.

The initial droplets in Figure 11 were processed using Photoshop CS6, and the droplet areas from the statistical maps were measured using Image Pro Plus 6.0. Then, the measured droplet areas were fitted to the same area as the circular droplet areas. The droplet diameters at X = −5 mm and X = −10 mm are shown in Figure 11a. The initial droplet size distribution was between 0.2 and 0.8 mm, and the initial droplet noticeably breaks up as it moves upward. Eventually, the droplet diameter decreased to 100 μm, and some droplets moved downstream from the recirculation zone. The melt column investigated in this study was 4 mm in diameter, and the fitted melt droplet diameter distribution was consistent with the conclusion obtained in the literature [19,30], namely that the droplet size after the main atomization is about 10 to 100% of the original size. The velocity profiles for positions X = −5 mm and X = −10 mm (Figure 11b) had an initial droplet velocity of 40 m/s. The velocity of the droplet as it approached the bottom of the delivery tube increased to 130 m/s. The droplet velocity at X = −5 mm was lower than at X = −10 mm, indicating that the initial droplet formation was significantly accelerated by turbulence during the movement at the boundary of the recirculation zone.

### 3.4. Effect of Gas Pressure on Powder Particle Size

**Figure 12:**
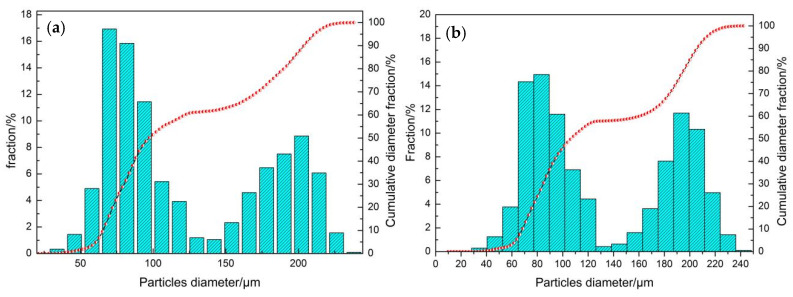

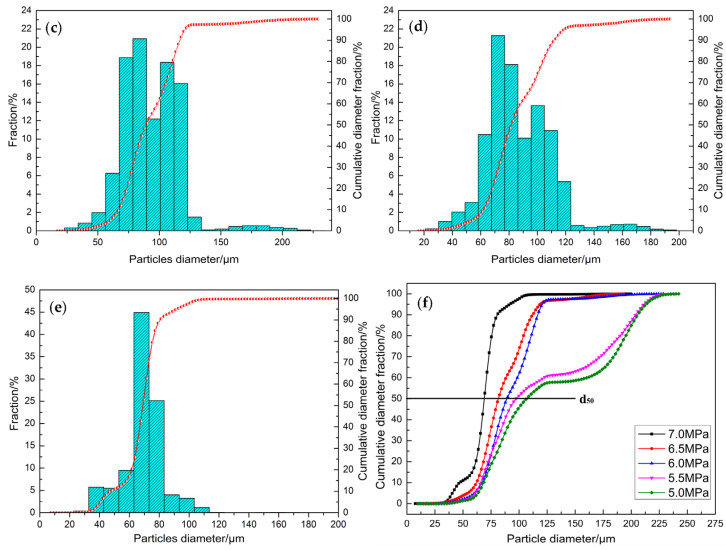
Particle size distributions of particles originating from increasing gas pressures. (**a**) 5.0 MPa; (**b**) 5.5 MPa; (**c**) 6.0 MPa; (**d**) 6.5 MPa; (**e**) 7.0 MPa; (**f**) Mass-based cumulative size distribution plots for five different pressures.

We investigated the effects of gas pressure at the nozzle inlets on powder particle size. The size distribution of droplet particles was obtained by monitoring the exit boundary of the physical model and counting the escaping particles, as shown in Figure 12. At an atomization pressure of 5 MPa (Figure 12a) and 5.5 MPa (Figure 12b), the particle size distribution of the titanium alloy powder is double-peaked, and powder in the range of 150 to 250 μm particle size accounts for more than 30%. The average particle size of the powder is larger, indicating that the secondary atomization process under this pressure is insufficient. The double-peaked phenomenon disappears at atomization pressures >5 MPa, and the particle size distribution shows a single-peaked normal distribution. With increasing gas pressure, the average particle size of the powder gradually decreases, and the powder exhibits a narrower particle size distribution. Figure 12f compares the cumulative particle size distribution of the powders under different simulated pressure conditions. The cumulative distribution curve shifts to the left as the atomization air pressure increases, i.e., the average particle size of the powders decreases. The median diameters (*d*_50_) of the powders are 110.5, 98.2, 90.3, 84.2, and 65.7 μm, respectively. Therefore, higher pressures may improve the efficiency of fine powder preparation.

## 4. Conclusions

We combined the computational fluid dynamics (CFD) approach and the volume of fluid (VOF) method to simulate primary breakup in an EIGA atomizer for titanium alloy powder production. In this paper, we present a general strategy to perform integral process modeling and coupled simulation of gas–melt interaction. We also study the effects of atomization and gas pressure on the powder particle size distribution. In general, the following conclusions were drawn regarding the atomization model:

Differential pressure caused an expansion wave cluster at the nozzle outlet end. Furthermore, the velocity increased and the pressure decreased, forming supersonic (>340 m/s) airflow with a maximum velocity of 660 m/s;

The formation of the recirculation zone was the direct driving force allowing atomization to proceed, which increased turbulence intensity and achieved higher atomization efficiency;

Recirculation was the main area where primary atomization occurred. The primary breakup process involved the expansion of the melt in recirculation to form a liquid membrane, which then peeled off the melt to form an umbrella-shaped structure. The liquid membrane continued to expand, forming an unstable liquid strip and finally breaking up to form the primary droplet. Under the studied operation conditions, the primary droplet size ranged between 200 and 800 μm, and the primary droplet velocity ranged between 40 and 130 m/s;

Appropriately increasing the gas pressure could effectively decrease the atomized powder particle size and the powder had a narrower particle size distribution.

## Figures and Tables

**Table 1:** Comparison between previous literature atomization models depending on the atomizing process generated by different gas atomization processes.

Reported by	Process	Condition	Model	Atomizing Process
Thompson et al. [11]	close-coupled gas atomiser	Sim	Euler–Lagrange(DPM)	primary atomization secondary atomization
Xing et al. [12]	pressure-swirl-gas-atomization	Sim	VOF + k-ω SST	primary atomization secondary atomization
Zeoli et al. [10]	VIGA	Sim	VOF +RSM	-
Wang et al. [20]	close-coupled gas atomiser	Sim + Exp	VOF + k-ω SST	primary atomization secondary atomization
Wang et al. [21]	VIGA	Sim + Exp	VOF + LES	primary atomization
Baraa et al. [22]	EIGA	Sim	RANS + k-ω SST	-
Wei et al. [3]	EIGA	Sim + Exp	-	-
Zeoli et al. [9]	gas atomization	Sim	RSM	secondary atomization

Sim: Simulation; Exp: Experiment.

**Table 2:** Physical properties of melt titanium (Ti) and argon (Ar) used in this study [22].

Thermophysical Properties	Density kg m^−3^	Condition	Surface Tension N m^−1^	Specific Capacity J kg^−1^ K^−1^	Thermal Conductivity Wm^−1^ K^−1^
titanium	4500	2.0 × 10^− 5^	1.588	812	25.8
argon	Ideal-gas	2.125 × 10^− 5^	-	520.64	0.0158

**Table 3:** Averaged values of the gas flow static pressures at the nozzle outlet computed for five different gas pressures.

Gas Pressure (MPa)	5.0	5.5	6.0	6.5	7.0
Static pressure (KPa)	−34.88	−36.62	−37.62	−38.32	−38.90
suction pressure (KPa)	−134.88	−136.62	−137.62	−138.90	−138.32

## Data Availability

Not applicable.
